# Is the Hedgehog Pathway Involved in the Pathophysiology of Schizophrenia? A Systematic Review of Current Evidence of Neural Molecular Correlates and Perspectives on Drug Development

**DOI:** 10.3390/cimb46060318

**Published:** 2024-05-27

**Authors:** Antonio Del Casale, Martina Nicole Modesti, Giovanna Gentile, Cecilia Guariglia, Stefano Ferracuti, Maurizio Simmaco, Marina Borro

**Affiliations:** 1 Department of Dynamic and Clinical Psychology and Health Studies, Faculty of Medicine and Psychology, Sapienza University of Rome, 00185 Rome, Italy; antonio.delcasale@uniroma1.it; 2 Unit of Psychiatry, Emergency and Admissions Department, Sant’Andrea University Hospital, 00189 Rome, Italy; 3 Department of Psychology, Faculty of Medicine and Psychology, Sapienza University of Rome, 00185 Rome, Italy; 4 Unit of Psychiatry, Mental Health Department, Santissimo Gonfalone Hospital, Local Health Service Roma 5, Monterotondo, 00015 Rome, Italy; 5 Department of Neuroscience, Mental Health and Sensory Organs (NESMOS), Faculty of Medicine and Psychology, Sapienza University, 00189 Rome, Italy; 6 Unit of Laboratory and Advanced Molecular Diagnostics, Sant’Andrea University Hospital, 00189 Rome, Italy; 7 Cognitive and Motor Rehabilitation and Neuroimaging Unit, Scientific Institute for Research, Hospitalization and Healthcare Fondazione Santa Lucia, 00179 Rome, Italy; 8 Department of Human Neuroscience, Faculty of Medicine and Dentistry, Sapienza University of Rome, 00185 Rome, Italy; stefano.ferracuti@uniroma1.it; 9 Unit of Risk Management, Sant’Andrea University Hospital, 00189 Rome, Italy

**Keywords:** Hedgehog proteins, Sonic Hedgehog, schizophrenia, antipsychotic agents, neuronal plasticity

## Abstract

Among the pathophysiological correlates of schizophrenia, recent research suggests a potential role for the Hedgehog (Hh) signalling pathway, which has been traditionally studied in embryonic development and oncology. Its dysregulation may impact brain homeostasis, neuroplasticity, and potential involvement in neural processes. This systematic review provides an overview of the involvement of Hh signalling in the pathophysiology of schizophrenia and antipsychotic responses. We searched the PubMed and Scopus databases to identify peer-reviewed scientific studies focusing on Hh and schizophrenia, following the Preferred Reporting Items for Systematic Reviews and Meta-Analyses statement, finally including eight studies, including three articles focused on patients with schizophrenia, two animal models of schizophrenia, two animal embryo studies, and one cellular differentiation study. The Hh pathway is crucial in the development of midbrain dopaminergic neurons, neuroplasticity mechanisms, regulating astrocyte phenotype and function, brain-derived neurotrophic factor expression, brain glutamatergic neural transmission, and responses to antipsychotics. Overall, results indicate an involvement of Hh in the pathophysiology of schizophrenia and antipsychotic responses, although an exiguity of studies characterises the literature. The heterogeneity between animal and human studies is another main limitation. Further research can lead to better comprehension and the development of novel personalised drug treatments and therapeutic interventions.

## 1. Introduction

Schizophrenia is a complex mental disorder characterised by various symptoms, with major ones including hallucinations, delusions, disorganised thinking, and cognitive deficits [1]. The aetiology and pathophysiology of this disorder are not yet fully understood, and current evidence suggests that genetic, environmental, psychological, and neurodevelopmental factors are involved in its pathogenesis within a multifactorial framework. Several traditional cellular signalling pathways, including glutamate, gamma-aminobutyric acid (GABA), and acetylcholine pathways, have been implicated in schizophrenia pathophysiology. Emerging research has highlighted new pathways potentially involved, including those related to gene expression in the 22q11 locus, the Disrupted in Schizophrenia 1 (DISC1) gene, Neuregulin 1/ Erythroblastic leukaemia viral oncogene homolog 4 (ErbB4) signalling, and components of the Protein kinase B/Glycogen synthase kinase-3 (Akt/GSK-3) pathway [2].

The Hedgehog (Hh) signalling pathway plays numerous roles in controlling cell proliferation, tissue organisation, stem cell maintenance, and development. In vertebrates, the primary cilium is the main centre for Hh signal transduction. In the absence of Hh, the Patched receptor (Ptch) localises to the cilium, inhibiting Smoothened activation (Smo). Glioma-associated oncogene proteins (Gli) are phosphorylated by Protein Kinase A (PKA), Casein Kinase I (CKI), and Glycogen Synthase Kinase 3 Beta (GSK3B) and partially degraded into the truncated repressor form of Gli (GliR). This repressor targets the cell nucleus, suppressing the transcription of Hh target genes.

**Figure 1:**
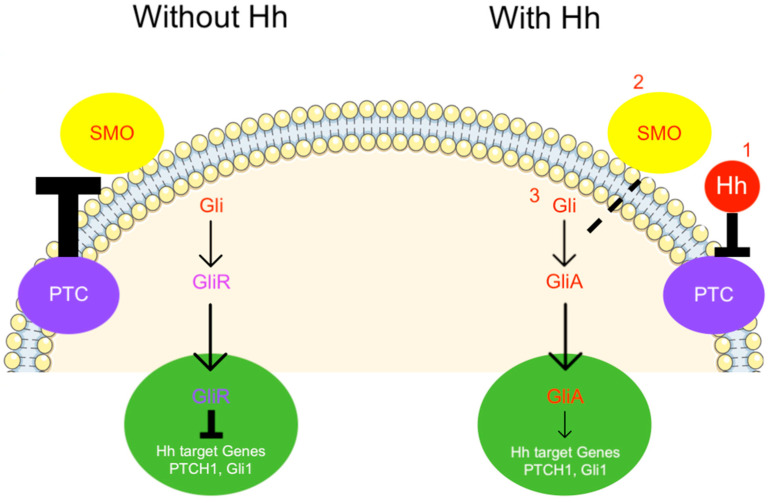
Main phases of Hh signalling pathway. Legend. Bold T: inhibition; Dashed line: activation; Arrow: downstream pathway activation.

In the presence of Hh, its receptor Ptch [3] disappears from the cilium, and the subsequent activation of Smo [4,5] leads to the translocation of the protein complex composed of Gli, Suppressor of Fused (Sufu), and Kinesin Family Member 7 (Kif7) to the cilium tip. At this point, Gli dissociates from the negative regulator Sufu, giving rise to the activator form of Gli (GliA), which accumulates in the nucleus, activating the transcription of Hh target genes [6]. We summarised these aspects in Figure 1. Other mechanisms are involved in this complex signalling pathway, which has been extensively reviewed [7,8].

Alterations in the Hh signalling pathway, initially studied for its role in embryonic development, tissue modelling, and oncogenesis, have recently been implicated as potentially involved in the neurodevelopmental abnormalities observed in schizophrenia since Hh also plays a role in neurogenesis by regulating neural cell proliferation and survival [7,9]. To date, there is evidence supporting intersecting signalling pathways with Hh, such as Wingless/Integrated (Wnt) [10,11], neurogenic locus notch homolog protein (Notch) [12], and dopamine pathways [13], indicating a network of interconnected signalling cascades contributing to schizophrenia pathophysiology. However, the exact mechanisms directly involving Hh must be thoroughly investigated.

Although the Hh signalling role becomes less predominant after embryogenesis, it continues to be active in stem cell-rich areas in the brains of adult mammals [14], exerting a particular influence on stem cell niches in the postnatal and adult brain’s subventricular zone by modulating precursor cells and controlling their proliferation. Such activity may be essential in brain homeostasis and neuroplasticity [15]. Investigating these functions could lead to advancements in treating brain injuries and neuroprotective and neuroregenerative therapies for adult neurological diseases and mental disorders [16,17,18]. Moreover, emerging evidence suggests a possible link between Hh signalling and various neuroinflammatory processes [19,20], paving the way for a multifaceted role of this pathway in the pathogenesis of neural alterations associated with schizophrenia risk.

Hypothesising that the Hh signalling pathway might be involved in the neuropathophysiology and antipsychotic drug response in schizophrenia, this review aims to provide an overview of the primary evidence regarding its involvement in schizophrenia and explore its implications in the neuropathophysiology of the disorder. Two other objectives are to highlight the potential therapeutic implications of targeting Hh signalling for schizophrenia treatment and to identify possible areas of future research to further understand this pathway’s involvement in schizophrenia and likely developments in pharmacology and personalised therapies.

## 2. Methods

We established the inclusion and exclusion criteria for articles to be incorporated into our review. Our inclusion criteria encompassed research involving human participants diagnosed with schizophrenia, irrespective of age, gender, ethnicity, or geographical location. We sought relevant studies focused on the Hh pathway in the neuropathophysiology of schizophrenia, including but not limited to molecular mechanisms associated with the Hh pathway correlated to schizophrenia or schizophrenia models, the potential role of Hh signalling components in schizophrenia pathogenesis, and the antipsychotic drug response. Additionally, the inclusion criteria for research type encompassed primary research studies, such as clinical trials, observational studies, cohort studies, case-control studies, and cross-sectional studies. We considered other inclusion criteria regarding outcome measures, encompassing studies reporting relevant outcomes such as changes in Hh pathway components/activity in schizophrenia patients or schizophrenia models, the correlation between Hh alterations and schizophrenia or schizophrenia-like symptomatology, and the influence of Hh modulation on antipsychotic drug efficacy or side effects. The final inclusion criterion was limited to peer-reviewed articles published in scientific journals.

Our exclusion criteria comprised studies focusing on mental disorders other than schizophrenia unless it was investigated as a comorbidity or comparison group. We excluded systematic reviews, meta-analyses, narrative reviews, and opinion articles, although these may serve as sources of references, which were not included as primary data. Additionally, studies needing peer review, such as conference abstracts, theses, dissertations, and preprints, were excluded. We also excluded studies that did not directly address the involvement of the Hh pathway in schizophrenia neuropathophysiology or antipsychotic drug response, as well as studies with insufficient data or unclear methodology to assess their relevance or quality.

After the definition of inclusion/exclusion criteria, we performed a search on PubMed (https://pubmed.ncbi.nlm.nih.gov/ (accessed on 23 March 2024)) and Scopus (https://www.scopus.com/ (accessed on 23 March 2024)) to identify peer-reviewed scientific studies focusing on Hh and schizophrenia. We followed a stepwise method to identify relevant experimental articles using the Preferred Reporting Items for Systematic Reviews and Meta-Analyses (PRISMA) method [21]. We did not follow a predetermined protocol or register this systematic review in public repositories for several factors. Firstly, our review included a combination of animal and human studies, reflecting different scopes of research that extended beyond human studies alone. The integration of animal research, which is valuable for comprehensive evidence synthesis, has other challenges in positioning with existing registration criteria, which do not consider mixed studies. Additionally, this review embraced studies with various research methodologies beyond the restrictions of randomised controlled trials (RCTs), further complicating its classification within standard registration criteria.

First, on 23 March 2024, we identified studies through a standard search with title/abstract specification using the terms “schizophrenia” and “Hedgehog”. In total, 23 articles were retrieved from PubMed. Subsequently, we employed the same search terms to retrieve 39 articles from the Scopus database. Second, we excluded articles focused on other signalling pathways, mental disorders other than schizophrenia, study protocols, commentaries, and reviews. Third, we searched for related studies on PubMed via the “Similar Articles” function and citation searching, and no additional relevant articles arose for inclusion in this review.

Based on these considerations, we considered 62 studies. After removing 17 duplicated articles, we excluded 19 reviews, 9 studies focused on Hh physiology, 2 articles unrelated to the topic, 2 focused on other pathways, 2 on different diagnoses, 2 short surveys, and 1 book chapter.

**Figure 2:**
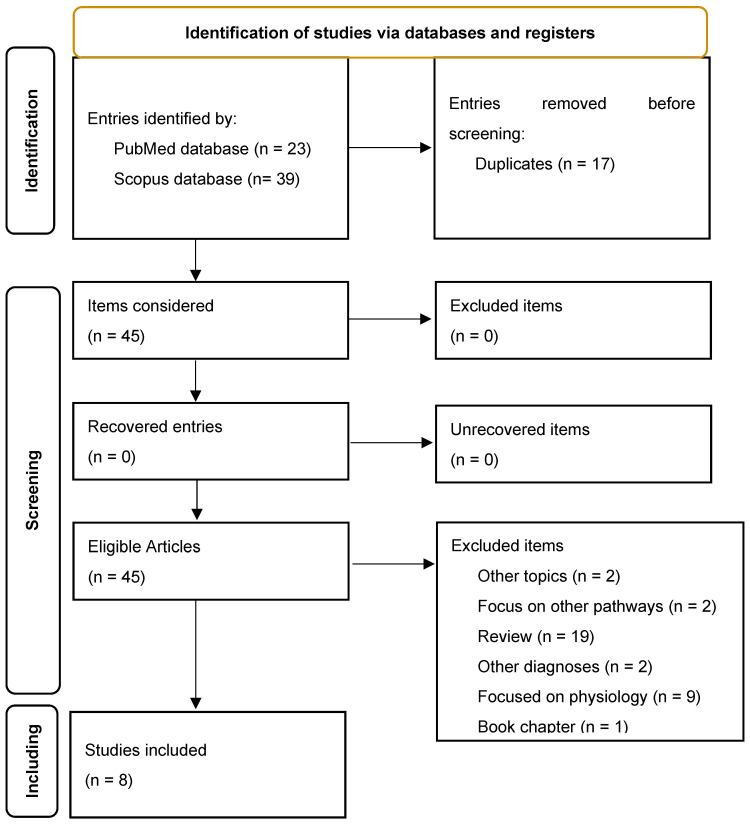
PRISMA flow diagram.

Finally, we included 8 articles [22,23,24,25,26,27,28,29] focusing on the involvement of the Hh pathway in the neuropathophysiology of schizophrenia and treatment response to antipsychotic drugs (see PRISMA flow diagram, Figure 2). 

The primary outcomes of this study concerned reported variants of genotypes and proteins involved in the Hh signalling and schizophrenia, models of schizophrenia, or antipsychotic response. For the assessment of the risk of bias in the included studies, we have used the Risk Of Bias In Non-randomised Studies of Interventions (ROBINS-I) tool [30] for human studies and the Centre for Laboratory Animal Experimentation (SYRCLE) tool [31] for animal studies.

## 3. Results

### 3.1. Included Studies

Our systematic review included 8 studies, encompassing cellular investigations on the impact of antipsychotics on the Hh pathway, animal experiments exploring schizophrenia models or genetic factors associated with the disorder and Hh signalling, and human research on Hh signalling involving patients diagnosed with schizophrenia.

Two cellular studies were focused on the Hh pathway and the effect of antipsychotic drugs. Lauth et al. [25] investigated the effects of clozapine, chlorpromazine, haloperidol, and imipramine on 7-dehydrocholesterol reductase (Dhcr7), an enzyme in cholesterol biosynthesis linked to Hh signalling, in National Institutes of Health 3 Thymidine kinase 3 (NIH3T3) cells and Sufu (−/−) mouse embryonic fibroblasts (MEFs). This study suggested that the regulation of Hh signalling could be an overlooked biological effect of therapeutics commonly used to manage schizophrenia and depression. Panizzutti et al. [28] treated Nerve Teratocarcinoma 2-Neuronal (NT2-N) cells with different antipsychotics, revealing decreased expression of genes in the Hh pathway, including glioma-associated oncogene homolog 2 (Gli2) and Cyclin D1 (CCND1), particularly with clozapine treatment. These studies have demonstrated that amisulpride, aripiprazole, clozapine, and haloperidol are among the most impactful antipsychotics on the Hh pathway.

Three animal studies were focused on the involvement of the Hh signalling in models of schizophrenia or on genes correlated with the risk of schizophrenia. Abdelfattah et al. [22] exposed male Wistar rats to social isolation and treated them with amisulpride or aripiprazole, finding that treatment reversed cognitive dysfunction induced by social isolation alongside changes in the Hh pathway and astrocyte activity. Boyd et al. [24] manipulated Sonic Hedgehog (Shh) signalling in zebrafish embryos, observing disruptions in disc1 expression and oligodendrocyte precursor cell specification, suggesting a potential link between aberrant Hh signalling and mental disorders. Mizoguchi et al. [27] analysed Shh signalling in Nerve Growth Factor (VGF)-overexpressing mice, finding no changes compared to wild-type mice.

Three human studies were focused on this topic. Betcheva et al. [23] conducted a GWAS involving 188 patients with schizophrenia or schizoaffective disorder and 376 healthy controls, revealing a significant association between schizophrenia and the intronic single nucleotide polymorphism (SNP) rs7527939 in the Hedgehog acyltransferase (HHAT) gene. A post-mortem study on patients with schizophrenia and controls by Reble et al. [29] identified a common variant in the Retinitis Pigmentosa GTPase Regulator Interacting Protein 1-Like (RPGRIP1L) gene associated with alternative splicing and Hh signalling, potentially influencing the pathophysiology of schizophrenia. Liu et al. [26] performed a Pathway-Wide Association Study (PWAS) on 5033 schizophrenia patients and 5332 controls from three different populations, identifying five pathways, including the Hh pathway, associated with schizophrenia across the analysed populations.

Two human studies [23,26] were conducted on patients with a diagnosis of schizophrenia, unrelated to organic aetiologies, based on the Diagnostic and Statistical Manual of Mental Disorders, 4th ed. [32], and the other post-mortem study [29] was conducted on seven patients with schizophrenia and four with bipolar disorder (unspecified diagnostic criteria).

The main characteristics of the included study are summarised in Table 1.

### 3.2. Causes of Heterogeneity among Study Results

Among human studies, all studies addressed an appropriate and focused question. One study had a high risk of bias, a post-mortem study that included two diagnoses in the sample. In all human studies, the analysed samples were selected from comparable populations in investigated outcomes. These are clearly defined in each study, and the diagnosis assessment method is reliable. In conclusion, the ROBINS-I risk of bias in the included human studies is generally moderate. Nevertheless, the results from these studies can be considered trustworthy.

Among animal studies, all studies addressed an appropriate and focused question. The SYRCLE risk of bias was generally low.

### 3.3. Causes of Heterogeneity among Study Results

The main heterogeneities are due to the study type, design, and methods. The included studies were on human subjects, animals, differentiated neuronal human cells, and differentiated cells from animal embryos. Among these, three pharmacological studies were conducted on an animal model of schizophrenia and animal embryos.

## 4. Discussion

### 4.1. Hh Signalling and Schizophrenia Pathophysiology

A Genome-Wide Association Study (GWAS) by Betcheva et al. demonstrated a significant association between schizophrenia and the intronic SNP rs7527939 in the Hh HHAT gene in a Bulgarian sample [23]. The precursor proteins of Hh must undergo amino-terminal palmitoylation by the HHAT protein before they can function as signalling molecules. HHAT overcomes the challenges of bringing together substrates with different physicochemical properties from opposite sides of the endoplasmic reticulum membrane within a membrane-embedded active site for catalysis [33]. The SNP rs7527939 is located in intron 2 of the HHAT gene, spanning 347,342 bp on chromosome 1q32.2. In vitro and in vivo studies have identified its function as an essential acyltransferase for the N-terminal palmitoylation of the Shh homologue product in embryonic and adult tissue precursor cells expressing the Hh gene.

In summary, HHAT controls the activity of Shh by catalysing its post-transcriptional modification, namely N-terminal palmitoylation [33,34]. The neurodevelopmental hypothesis of schizophrenia suggests that alterations in the integrity of interneural networking, as part of disrupted neurodevelopment, underlie the disease with a long-term consequence of neuronal vulnerability to harmful environmental factors [35]. Errors in neuronal cell migration and interaction during embryonic development may stem from altered activity in the Hh pathway [23]. Shh, whose activity is controlled by HHAT, plays an essential role in inducing the differentiation of dopaminergic neuroblasts and in cell migration towards specific sites of dopaminergic neurons in the mammalian brain. It has been proposed that the regulation of HHAT–Shh could be involved in the complex multifactorial pathophysiology of schizophrenia, also based on dysregulations in the neurodevelopment and neurotransmission of dopaminergic circuits. However, it is not yet clear whether specific genotypes of rs7527939 have any functional effect on HHAT or the etiopathogenesis of schizophrenia [23]. In addition to the biological role of the HHAT gene, the region of its chromosomal localisation has been suggested as a candidate region for schizophrenia by several genetic linkage studies and cytogenetic findings. Some studies have identified different genes or nearby chromosomal regions potentially involved (mainly 1q32.2-q4) in the pathophysiology of schizophrenia, although not always with consistent results [36,37,38].

The RPGRIP1L gene encodes a ciliary protein crucial for brain development processes, including Shh signalling, neural tube formation, and the formation of left–right asymmetry. This gene has been implicated in telencephalic development, with complex functions of primary cilia in telencephalic morphogenesis through region-specific modulation of the Hh pathway [39], hypothalamic arcuate neuron development [40], telencephalic developmental disorders [41], and pathophysiology of bipolar disorder and high body mass index [42].

In line with this background, a post-mortem study on 7 patients with schizophrenia, 4 with bipolar disorder, and 79 controls showed variation in the levels of two transcripts of the RPGRIP1L gene among the groups, possibly correlated with the genotypes of rs8050354 and rs7203525. When these two isoforms were quantified in each brain tissue sample using Droplet Digital Polymerase Chain Reaction, the RPGRIP1L transcript with exon 20 spliced out was significantly decreased in the dorsolateral prefrontal cortex and hippocampus of individuals with schizophrenia and bipolar disorder risk alleles rs8050354(T) and rs7203525(T) [29]. These findings support the hypothesis of Hh signalling involvement in altered neurodevelopment in schizophrenia.

Furthermore, the Hh signal has recently been identified as a promoter of disc1 protein expression in the zebrafish brain [24]. This discovery raises the hypothesis that Hh could be involved in the pathophysiology of schizophrenia through its role in disc1 expression. Previous studies have established that DISC1 has been extensively studied as a genetic risk factor for mental illnesses. Although no common variants at the DISC1 locus associated with schizophrenia have been found in broader populations, this does not exclude the possibility that DISC1 indicates central biological pathways for mental health issues. Observing a loss of disc1 expression in smoothened mutants lacking Hh signal transduction and elevated expression in patched mutants with constitutive Hh signalling activation suggests its significant involvement in regulating disc1 and, consequently, in possible mechanisms underlying schizophrenia [24].

The genetic evidence regarding the association between DISC1 and schizophrenia shows inconsistencies [43]. However, there is evidence that the D2R-DISC1 protein complex and associated proteins are altered in schizophrenia and that such alterations regress with treatment with atypical antipsychotics [44]. In this regard, DISC1 may impact developmental pathways and pathophysiological mechanisms related to schizophrenia, among which modifications in the Hh signalling pathway appear to be an exciting candidate deserving further investigation.

In line with this hypothetical involvement, a recent Pathway-wide Association Study (PWAS) has unearthed five commonly implicated pathways linked to schizophrenia susceptibility across three distinct ancestral populations [26]. While GWASs have highlighted the polygenic nature of schizophrenia, with numerous alleles associated with increased vulnerability, the utility of any single allelic marker remains limited, prompting a shift towards considering schizophrenia as a disorder influenced by multiple pathways. To empirically explore this notion, a PWAS encompassing 255 Kyoto Encyclopedia of Genes and Genomes pathways was conducted among 5033 individuals diagnosed with schizophrenia and 5332 unrelated healthy controls from European-American (EA), African American (AA), and Han Chinese (CH) populations. The results unveiled 103, 74, and 87 pathways associated with schizophrenia susceptibility in EA, CH, and AA populations. Noteworthily, Hh signalling was among the pathways (serotonergic synapse, ubiquitin-mediated proteolysis, adipocytokine signalling, and renin secretion) that exhibited shared relevance across all three populations. The sets of single-nucleotide polymorphisms representing these pathways were found to be enriched for regulatory functions. These findings empirically bolster the notion of schizophrenia as a poly-pathway disorder characterised by both genetic and pathway heterogeneity [26]. In the context of this evidence, it has been emphasised that Hh signalling interacts with the ubiquitin-mediated proteolysis pathway [45] and that the most characterised ligand of the pathway, Shh, is widely distributed in the adult human central nervous system and regulates the generation of functional synaptic contacts [46,47], thus being involved in neuroplasticity phenomena. Furthermore, oligodendrocyte and white matter abnormalities have been widely documented in schizophrenia, and the importance of Hh signalling in oligodendrocyte development is well documented [48,49]. Therefore, it could be hypothesised that dysfunction in Hh signalling in the midbrain may adversely influence dopaminergic neuron development, potentially increasing susceptibility to schizophrenia, in the context of the ‘two-hit’ hypothesis of schizophrenia, which suggested that disruption of the Hh pathway during brain development may predispose the central nervous system to a pathological response in the event of a second hit in adulthood [35].

### 4.2. Hh Pathway and Antipsychotics

Existing antipsychotic medications target dopamine receptors, and it has long been discovered that Hh signalling is essential for developing midbrain dopaminergic neurons [50]. A recent study investigated the effects of the antipsychotics amisulpride and/or aripiprazole on the Hh signalling pathway and its relation to cognitive functions and neurogenesis in a rat model of schizophrenia. Behavioural, biochemical, and histopathological tests were conducted on male Wistar rats and allocated to control, socially isolated, amisulpride, and aripiprazole-treated groups. Cognitive dysfunction, evidenced in socially isolated rats, was associated with disorganised Hh signalling pathway protein levels and increased astrocyte activity. Treatment with amisulpride and/or aripiprazole reversed these changes, enhancing Hh pathway components, dopamine-1 receptors, and BDNF and decreasing astrocyte activity and inflammatory markers. These findings suggest a favourable role for amisulpride and aripiprazole in regulating the expression of D1 receptors and BDNF and the Hh signalling pathway activation, leading to cognitive and neurogenesis improvements [22]. Amisulpride and/or aripiprazole were found to stimulate the Hh signalling pathway, as their administration resulted in a significant increase in concentrations of Patched (Ptch), Glioma-associated oncogene homolog 1 (Gli1), BDNF levels, and D1 receptor mRNA, along with a significant decrease in Glioma-associated oncogene homolog 3 (Gli3) concentrations.

Additionally, their administration was associated with reduced neuroinflammation, as indicated by decreased levels of interleukin-1 beta (IL-1β), interleukin-6 (IL-6), and tumour necrosis factor-alpha (TNF-α), whose effects were correlated with Hh levels. These findings were demonstrated in animal models of schizophrenia with associated cognitive dysfunction induced by a social isolation protocol [22]. Furthermore, Hh signalling has been associated with neurogenesis, as it enhances BDNF secretion, which has been considered a neuropathophysiological correlate of psychosis since reduced BDNF levels have been reported in the serum of patients with first-episode psychosis compared to healthy subjects [51,52].

However, even though VGF is a neuropeptide precursor induced by BDNF and elevated levels of VGF have been detected in the prefrontal cortex and cerebrospinal fluid of individuals with schizophrenia, a study conducted on VGF-overexpressing mice exhibiting schizophrenia-like behaviours revealed no alterations in the Hh pathway [27].

Another central aspect of Hh is its astrocyte function and phenotype regulation, as astrocytes are highly responsive to Shh. Therefore, the physiological maintenance of this pathway can reflect the arrangements of neuroprotective mechanisms in the central nervous system [53,54]. Consistent with this evidence, impairment and dysregulated expression of the Sonic Hh and alterations in BDNF and astrocytes have been associated with various neurodevelopmental disorders, including schizophrenia [51,55,56]. Additionally, post-mortem studies on patients with schizophrenia have highlighted altered astrocytic activity, reporting significant changes in astrocyte morphology, density, and dysregulated expression of astrocyte-defining cellular markers associated with a significant increase in the levels of glial fibrillary acidic protein (GFAP) [57,58]. These results may be linked to studies demonstrating that social isolation correlates with the dysregulation of GFAP, BDNF, and D1 receptors, which are highly expressed in the prefrontal cortex and involved in controlling working memory and cognitive functions [22]. Reduced activation of D1 receptors has been associated with cognitive dysfunction and negative symptoms of schizophrenia [59]. The connection between the Sonic Hh pathway and D1 receptor functionality has been recognised and supported by the fact that the activation of this pathway increases glutamate, which induces N-methyl-D-aspartate (NMDA) receptors, which in turn influence the density of D1 receptors [60]. The activation of NMDA receptors recruits D1 receptors to the plasma membrane and shifts the balance of dopamine signalling toward the D1 receptors and away from the D2 receptors [61]. This evidence related to the Hh signalling pathway could be incorporated into the context of the glutamatergic hypothesis of schizophrenia [62].

It has been highlighted that antipsychotics may induce an in vivo modification in the transcription of the DHCR7 gene, consequently altering the formation of the enzyme Dhcr7, responsible for the final step in cholesterol production, which is associated, in turn, with the activation of the Hh signalling pathway [63,64]. In this context, it has been noted that the Gli antagonist 61 (Gant61), a small-molecule antagonist of Gli transcription factors involved in Hh signalling, shares structural similarities with AY9944, an inhibitor of Dhcr7. Treatment with the diamine Gant61, AY9944, or overexpression of Dhcr7 attenuates Hh signalling. Antipsychotic treatments have increased Hh signalling activation with Gli1 activation in vitro and in vivo [63]. Correlating with the in vivo downregulation of Dhcr7, Hh signalling following clozapine, chlorpromazine, or haloperidol administration is increased with upregulation of Gli1 expression and activity, further supporting the existence of an inverse correlation between Dhcr7 and Gli1 levels [63].

The evidence presented may be linked to reported myelin aberrations in schizophrenia. Oligodendrocyte-derived myelin membranes in the central nervous system are highly enriched with various lipids, including cholesterol, phospholipids, and glycosphingolipids. This observation underscores the importance of considering lipid homeostasis as a relevant target for studying the genetics, pathophysiology, and drug treatment of schizophrenia [65]. In a similar context, the involvement of Hh signalling may be related to both the pathophysiology of schizophrenia (such as changes in myelin homeostasis, axonal development, and neural plasticity in general), mechanisms of action of antipsychotic drugs (such as the induction of NMDA receptors, the shift of dopamine towards D1 receptors, and Dhcr7 modifications), and potential pharmacological side effects.

Furthermore, drugs used to treat schizophrenia also modulate the Hippo signalling pathway, known to interact with Hh signalling. Treatment with amisulpride, aripiprazole, clozapine, quetiapine, or risperidone reduced genes associated with the Hippo signalling pathway. These drugs also modulated genes involved in interacting pathways, suggesting a potential reduction in pro-inflammatory signalling. Connectivity map analysis identified compounds that may act similarly to these antipsychotics, including nine compounds whose mechanisms are, in turn, associated with a reduction in nuclear factor kappa-light-chain-enhancer of activated B cells (NFκB) signalling, namely ursolic acid, carbimazole, NS-398, sodium phenylbutyrate, furazolidone, iloprost, ergocalciferol, clozapine, and triflupromazine, revealing potential targets for drug repurposing [28]. In particular, the expression of proteins coded by genes in the Hh signalling pathway was decreased overall by clozapine. This drug significantly reduced the expression of CCND1, cyclin D2 (CCND2), and Gli2, which are involved in cellular processes, including cell proliferation, differentiation, and development [28]. This effect may align with the evidence of cerebral morphometric changes induced by clozapine [66]. The mechanisms by which clozapine might modulate the Hh signalling pathway are not fully understood, and clarifying these aspects could have significant clinical implications for the treatment of schizophrenia.

Furthermore, evidence suggests that the Hh and Wnt pathways are interconnected. For instance, the Hh signalling pathway attenuates Wnt activity through activated Secreted Frizzled Related Protein 1 (SFRP1), while the Wnt/β-catenin pathway provides feedback to regulate Hh activity through the transcriptional regulation of Gli3 [67]. Based on this premise, antipsychotics affecting a specific pathway may subsequently influence the other pathway.

**Figure 3:**
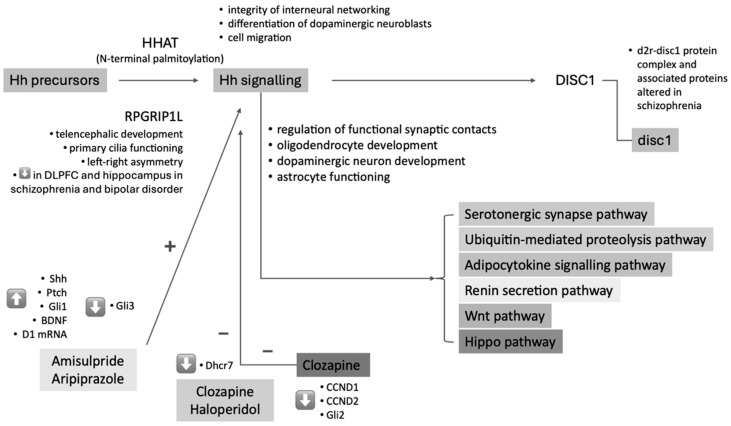
The main body of evidence of the involvement of Hh signalling in the pathophysiology of schizophrenia and the mechanism of action of antipsychotics. Legend: BDNF: Brain-Derived Neurotrophic Factor; CCND1: Cyclin D1; CCND2: Cyclin D2; D1: Cyclin D1; d2r: Dopamine D2 receptor; DISC1: Disrupted in Schizophrenia 1 gene; disc1: Disrupted in Schizophrenia 1 protein; Dhcr7: 7-Dehydrocholesterol Reductase; DLPFC: Dorsolateral Prefrontal Cortex; Gli1: Glioma-Associated Oncogene Homolog 1; Gli2: Glioma-Associated Oncogene Homolog 2; Gli3: Glioma-Associated Oncogene Homolog 3; HHAT: Hedgehog Acyltransferase; Hh: Hedgehog; mRNA: Messenger Ribonucleic Acid; Ptch: Patched; RPGRIP1L: Retinitis Pigmentosa GTPase Regulator Interacting Protein 1-Like; Shh: Sonic Hedgehog; SUFU: Suppressor of Fused; Wnt: Wingless/Integrated.

We have highlighted in Figure 3 the main involvements of the Hh pathway in the pathophysiology of schizophrenia and the mechanism of action of some antipsychotics. Considering the limited number of studies on the subject, further research is needed to elucidate the specific mechanisms involved and to determine whether targeting the Hh pathway could be a viable therapeutic strategy for schizophrenia.

### 4.3. Limitations

The limited number of studies included could reduce the generalizability of reported evidence and the detection of actual effects. Additionally, combining findings from animal, embryo, and human studies requires cautious interpretation due to potential species and developmental differences. However, because of the limited number and heterogeneity of the current pioneering studies in this area, our objective is to assess the potential role of the Hedgehog pathway in the neuropathophysiology of schizophrenia. These insights can potentially advance diagnostic procedures, inform clinical management strategies, and facilitate drug discovery efforts in this field.

## 5. Conclusions

In summary, the multifaceted role of Hh signalling in the pathophysiology of schizophrenia may either consist of its direct involvement or include intricate interactions with various cellular signalling pathways, such as Wnt, Hippo, dopamine, and NFκB. These interactions highlight the complexity of the molecular framework related to schizophrenia and likely the underlying neurodevelopmental alterations, emphasising the potential importance of Hh pathway dysregulation in the disorder’s pathophysiology. Furthermore, recent evidence of the ability of antipsychotic drugs to modulate the Hh pathway underscores the therapeutic relevance of targeting this pathway in the treatment of schizophrenia. In addition to their best-known mechanisms of action, these drugs may exert additional effects on cellular signalling pathways, enabling another precision approach to address the various biological mechanisms implicated in schizophrenia.

Based on the limited evidence, the potential of modulating the Hh pathway in mitigating neuroinflammation and promoting neuronal plasticity appears to be a relevant aspect implicated in the pathophysiology of schizophrenia. By targeting neuroinflammatory processes and neuronal plasticity, modulation of the Hh pathway could be a promising strategy for treating the molecular and cellular correlates of schizophrenia. Future studies in this field could increase knowledge of the intricate interplay between Hh signalling and the pathophysiology of schizophrenia, paving the way for the development of innovative and precise therapeutic strategies. Furthermore, elucidation of the molecular underpinnings of Hh dysregulation in specific subtypes of schizophrenia, such as those possibly associated with neuroinflammation, could facilitate the identification of personalised therapeutic approaches aimed at improving outcomes and enhancing the quality of life of affected individuals.

## Figures and Tables

**Table 1:** The main characteristics of the included studies.

Study	Type of Study	Sample	Main Results
Abdelfattah et al., 2023 [22]	Animal study	60 male Wistar rats grouped in controls and socially isolated, treated with amisulpride or aripiprazole.	Social isolation-induced cognitive dysfunction alongside disorganised Shh-pathway protein levels and increased GFAP-stained astrocytes. Treatment reversed these effects, evidenced by elevated Shh, Ptch-1, Smo, GLI-1, dopamine-1 receptors, and BDNF, and decreased GLI-3 protein, GFAP immune reactivity in astrocytes, and inflammatory markers.
Betcheva et al., 2013 [23]	GWAS on 554,496 SNPs	188 patients with schizophrenia and 376 controls	Significant association between schizophrenia and the intronic SNP rs7527939 in the HHAT gene.
Boyd et al., 2015 [24]	Animal study	Genetic and chemical modulation of Shh signalling on disc1 expression in the zebrafish embryo and double-labelling methods to characterise disc1-expressing cells in the hindbrain	The robust expression of disc1 in Olig2-positive midline progenitor cells is disrupted in Smo mutants, with Cyclopamine treatment mirroring the effects of disc1 knockdown on oligodendrocyte precursor cells specification by blocking disc1 expression.
Lauth et al., 2010 [25]	Animal study	NIH3T3 cells, ShhL2 cells, and Sufu (−/−) MEFs	Clozapine, chlorpromazine, haloperidol, and the antidepressant imipramine modulated DHCR7, influencing Hh signalling both in vitro and in vivo.
Liu et al., 2017 [26]	PWAS	5033 patients with schizophrenia and 5332 controls across EA, AA and CH populations	Five pathways (serotonergic synapse, ubiquitin-mediated proteolysis, Hh, adipocytokine, and renin secretion) were associated with schizophrenia and shared among the three populations. Their respective SNP sets showed enrichment for SNPs with regulatory functions, indicating potential roles in genetic regulation across diverse populations.
Mizoguchi et al., 2019 [27]	Animal study	VGF-overexpressing mice	There were no changes in Shh signalling between Wild-type and VGF-overexpressing mice.
Panizzutti et al., 2021 [28]	Genome-wide mRNA expression study	NT2-N cells were treated with amisulpride, aripiprazole, clozapine, quetiapine, and risperidone.	Expression of genes in the Hh signalling pathway was decreased overall by clozapine and tended to be reduced by aripiprazole. Clozapine significantly reduced the expression of Gli2 and DCCN1 and showed a trend towards reducing the expression of CXCR4 and CCND1.
Reble et al., 2019 [29]	Post-mortem study	7 patients with schizophrenia, 4 with bipolar disorder, and 79 controls	A common variant in RPGRIP1L, known as rs7203525 and involved in Hh signalling, has been identified to influence alternative splicing, increasing the inclusion of exon 20 of RPGRIP1L. A minigene assay combined with in vitro mutagenesis confirmed that this alternative splicing is directly linked to the alleles of this variant. The predominant RPGRIP1L isoform expressed in adult brains typically lacks exon 20.

Table legend. A.A.: African American; B.D.N.F.: Brain-Derived Neurotrophic Factor; CH: Han Chinese; CCND1: Cyclin D1; CXCR4: C-X-C Chemokine Receptor Type 4; DHCR7: 7-Dehydrocholesterol Reductase; DCCN1: Discoidin Domain Receptor Tyrosine Kinase 1; E.A.: European-American; G.F.A.P.: Glial Fibrillary Acidic Protein; Gli1: Glioma-Associated Oncogene Homolog 1; Gli2: Glioma-Associated Oncogene Homolog 2; GWAS: Genome-Wide Association Study; Hh: Hedgehog; H.H.A.T.: Hedgehog Acyltransferase; M.E.F.: mouse embryonic fibroblast; mRNA: Messenger Ribonucleic Acid; NIH3T3: National Institutes of Health 3 Thymidine kinase 3; NT2-N; Nerve Teratocarcinoma 2-Neuronal; Olig-2: Oligodendrocyte Transcription Factor 2; Ptch-1: Patched-1; P.W.A.S.: Pathway-Wide Association Study; RPGRIP1L: Retinitis Pigmentosa GTPase Regulator Interacting Protein 1-Like; Shh: Sonic Hedgehog; ShhL2: Sonic Hedgehog Light 2; Smo: Smoothened; SNP: Single Nucleotide Polymorphism; Sufu: Suppressor of Fused; V.G.F.: Nerve Growth Factor.

## Data Availability

Not applicable.
